# Radiomics-machine learning model for predicting invasiveness of subcentimeter subsolid lung adenocarcinoma: a validation study with external cohort and SHAP interpretability

**DOI:** 10.3389/fonc.2026.1668102

**Published:** 2026-03-26

**Authors:** Wenfeng Feng, Ruiting Chang, Tiezhi Li, Xiaolong Wang, Zhihong Gao, Xu Yang, Yuling Yin, Yuqiang Zuo

**Affiliations:** 1 Medical Imaging Center, The Second Hospital of Hebei Medical University, Shijiazhuang, Hebei, China; 2 Department of Imaging Center, Harrison International Peace Hospital, Hengshui, Hebei, China; 3 Thoracic surgery, The Second Hospital of Hebei Medical University, Shijiazhuang, Hebei, China; 4 Information Center, The Second Hospital of Hebei Medical University, Shijiazhuang, Hebei, China; 5 Physical Examination Center, The Second Hospital of Hebei Medical University, Shijiazhuang, Hebei, China

**Keywords:** decision curve analysis, explainable artificial intelligence, lung adenocarcinoma, machine learning, radiomics

## Abstract

**Background:**

Preoperative discrimination of invasive adenocarcinoma (IAC) from pre-invasive lesions in subcentimeter subsolid nodules (SSNs) remains challenging using conventional computed tomography (CT). We aimed to develop and validate an interpretable radiomics-machine learning (ML) model for predicting invasiveness by leveraging SHapley Additive exPlanations (SHAP).

**Methods:**

In this two-center retrospective study, 177 patients from Hospital 1 (training and internal validation) and 83 patients from Hospital 2 (independent external validation) with surgically confirmed lung adenocarcinoma manifesting as SSNs (≤1 cm) were enrolled. Radiomic features were then extracted from preoperative CT using the uAI Research Portal. Following a reproducibility assessment (intraclass correlation coefficient >0.75), the minimum Redundancy Maximum Relevance (mRMR) and Least Absolute Shrinkage and Selection Operator (LASSO) regression were applied to select the most predictive features. Three ML classifiers: logistic regression (LR), random forest (RF) and support vector machine (SVM) were trained and validated using a 7:3 cohort split, and the best-performing model was further evaluated in the external validation cohort. Model performance was evaluated by the area under the receiver operating characteristic curve (AUC), sensitivity, specificity, F1 score, calibration, and decision curve analysis (DCA). SHAP analysis was employed to provide global and local model interpretability.

**Results:**

A set of ten radiomic features was selected to predict invasiveness (IAC prevalence: 44.6%). The LR model demonstrated optimal performance during internal validation (AUC: 0.842; sensitivity: 79.2%; specificity: 73.3%; F1 score: 0.745) and exhibited superior generalizability compared to both the RF and SVM models. In the external validation cohort, the LR model maintained robust diagnostic performance, with an AUC of 0.778 (95%CI: 0.673-0.862), confirming its cross-institutional generalizability. The DCA and PRC curves further confirmed its clinical utility and stability across different institutions. SHAP analysis identified wavelet_HLL_glszm_LowGrayLevelZoneEmphasis (an indicator of necrosis), original_shape_Flatness (reflecting morphological irregularity), and log_firstorder_LoG.Minimum (suggestive of air-trapping) as top predictors of invasiveness. Decision curve analysis confirmed the model’s superior clinical utility over empirical management strategies.

**Conclusion:**

The developed radiomics-LR model robustly predicts invasiveness in subcentimeter SSNs and provides biologically plausible explanations through SHAP. Its balanced performance and inherent interpretability support its potential integration into clinical workflow to aid in surgical decision-making.

## 1 Introduction

Lung cancer is the most common malignant tumor and the leading cause of cancer-related mortality worldwide (1). Non-small cell lung cancer (NSCLC) constitutes approximately 85% of all lung cancer cases, with adenocarcinoma being the most prevalent subtype (2). The widespread adoption of low-dose computed tomography (LDCT) for lung cancer screening has substantially increased the detection of pulmonary nodules, particularly subcentimeter subsolid nodules (SSNs) (3, 4). SSNs, encompassing both pure ground-glass nodules and part-solid nodules, exhibit heterogeneous biological behaviors, ranging from pre-invasive lesions (e.g., atypical adenomatous hyperplasia) to invasive adenocarcinomas (IAC) (5).

Accurately characterizing these nodules is critical, as stage I NSCLC (including stage IB disease) demonstrates remarkable heterogeneity in prognosis. Even after complete resection, the 5-year overall survival for stage IB NSCLC is approximately 73%, with recurrence rates as high as 18-29% (6, 7). Accurate preoperative prediction of invasiveness in SSNs remains a diagnostic challenge, as their imaging characteristics frequently overlap across different pathological subtypes. Conventional radiological assessment depends on subjective morphological characteristics such as size, margin, and solid component proportion, which often suffer from limited diagnostic precision (8). Furthermore, the role of adjuvant therapy in early-stage NSCLC like stage IB remains a subject of debate (9), highlighting the need for better tools to identify high-risk patients who might benefit from adjuvant therapy or more extensive resection.

Radiomics, which is an emerging field in quantitative imaging analysis, enables high-throughput extraction of minable data from medical images, capturing subtle patterns imperceptible to the human eye (10). When combined with machine learning (ML), radiomics facilitates the development of robust predictive models for nodule characterization (11). Recently, studies have endeavored to predict the invasiveness of subsolid nodules using advanced methodologies. For instance, Zuo et al. developed a combined nomogram integrating deep-learning-assisted CT texture features, achieving robust performance (12). Similarly, Li et al. demonstrated the superior performance of a stacking ensemble machine learning model that combines radiomic signatures with clinical-radiological features (13). These studies underscore the potential of quantitative imaging analysis. However, the specific challenge of preoperatively predicting invasiveness in SSNs, which are particularly elusive on conventional CT, remains less explored. Furthermore, the comprehensive integration of explainable AI (XAI) techniques, such as SHAP, is still needed to provide transparent, biologically plausible explanations for model predictions in this specific context (14). Therefore, this study aims to develop an interpretable radiomics-machine learning model specifically for SSNs, with a strong emphasis on leveraging SHAP analysis to elucidate the model’s decision-making process and enhance clinical trustworthiness.

## 2 Materials and methods

### 2.1 Patient cohorts

**Figure 1:**
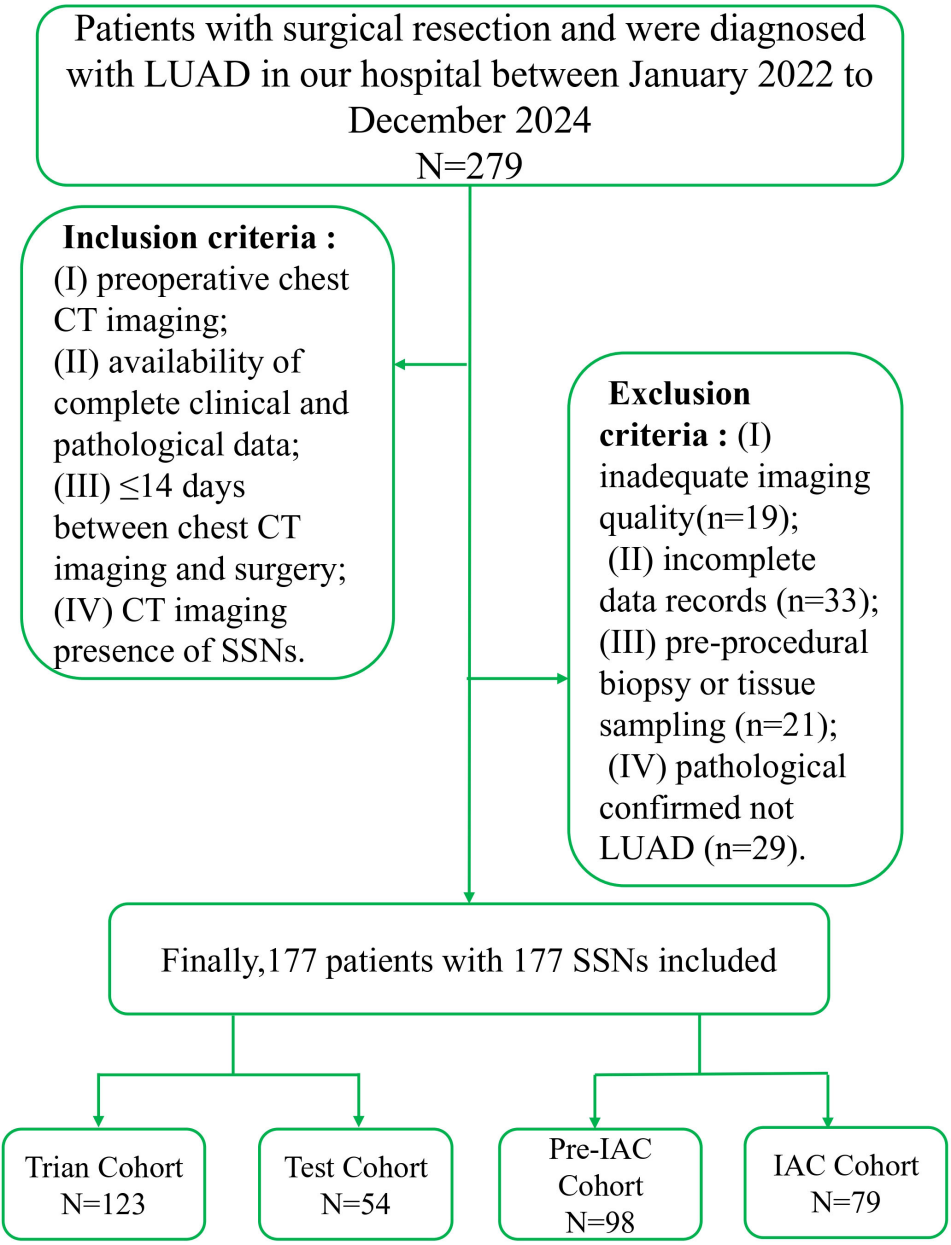
Flowchart of study participant enrollment.

A retrospective cohort study was conducted at our institution involving 177 consecutive patients with pathologically confirmed lung adenocarcinoma (LUAD) who underwent surgical resection. Inclusion criteria included: (I) preoperative chest CT imaging, (II) availability of complete clinical and pathological data, (III) ≤14-day interval between preoperative chest CT imaging and surgery, and (IV) radiologically confirmed presence of SSNs on CT imaging. Exclusion criteria were: (I) inadequate CT image quality, (II) incomplete data records, (III) history of neoadjuvant chemotherapy or radiotherapy, and (IV) non-LUAD pathological diagnosis. The study was approved by the Institutional Ethics Committee of the Second Hospital of Hebei Medical University (approval number: 2023-R384) and conducted in accordance with the Declaration of Helsinki. Written informed consent was waived due to the retrospective design. Patients were randomly stratified into training (70%, n=123) and internal validation (30%, n=54) cohorts using a 7:3 ratio. A flowchart detailing patient enrollment is presented in **Figure 1**. To evaluate the generalizability of the model, an independent external validation cohort was collected from Harrison International Peace Hospital between 2023 and 2024. A total of 83 patients with subcentimeter SSNs were enrolled using the same inclusion and exclusion criteria as the primary cohort.

### 2.2 CT image acquisition

All participants underwent **preoperative standard-dose** chest CT scans within 2 weeks before surgery. Standard-dose protocols were employed to ensure optimal image quality for the assessment of subcentimeter lesions. During CT acquisition, patients were positioned supine with arms elevated above their heads. The scanning range extended from the thoracic inlet to the adrenal gland region. Helical CT continuous scanning was performed during deep inspiration breath-hold. The CT systems included a GE Optima 660 64-slice helical CT (United States) and a Philips iCT 256-slice helical CT (Netherlands). To minimize potential confounding factors arising from inter-scanner variability, the external validation cohort from Hospital 2 was specifically derived from patients scanned using the same CT platforms as the primary cohort. All CT examinations were performed following the manufacturers’ default clinical protocols, identical to those described for Hospital 1: (tube voltage 120 kV, tube current 100–300 mA with adaptive modulation technology, matrix size 512×512, slice thickness 5.0 mm, and spacing 5.0 mm, with reconstruction layer thicknesses of 1.0 mm or 1.25 mm). Thin-section reconstructions (<1.5 mm thickness) using high-resolution and standard algorithms generated lung and mediastinal window images respectively. Notably, all CT images used for radiomic feature extraction were reconstructed using sharp kernels (Lung window algorithms) to optimize the visualization of fine-grained nodular structures. To minimize the impact of reconstruction-induced noise on feature extraction, a standardized preprocessing workflow (including resampling and normalization) was applied as described in Section 2.3. Image analysis utilized: lung window settings (width 1,500 HU, level 600 HU) and mediastinal window settings (width 350 HU, level 40 HU).

### 2.3 ROI segmentation and radiomic feature extraction

**Figure 2:**
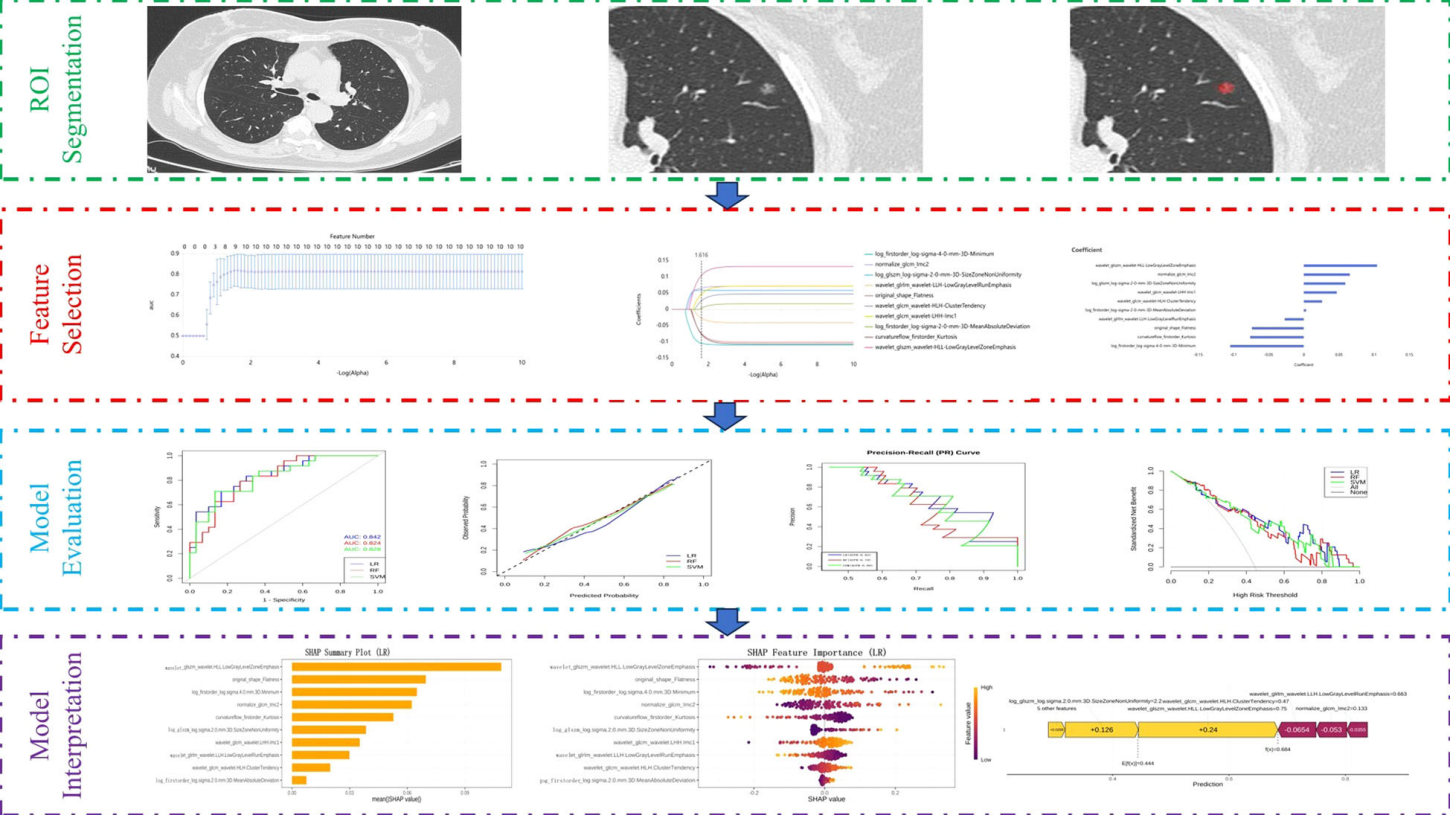
Workflow of the radiomic analysis pipeline.

The overall radiomics workflow is illustrated in **Figure 2**. The integrated workflow, including region of interest (ROI) segmentation, radiomic feature extraction, feature selection, and ML model development. Radiomic features were extracted using the uAI Research Portal (https://urp.united-imaging.com/; United Imaging Intelligence) (15). The extraction engine of this platform is based on the PyRadiomics framework, which is compliant with the Imaging Biomarker Standardization Initiative (IBSI) standards, ensuring the reproducibility and standardization of the extracted biomarkers. Processing commenced with automated ROI outlining by the uAI platform, which was subsequently manually revised, and finally confirmed by a single board-certified radiologist with 15 years of experience in thoracic CT. Delineated ROIs encompassed the entire tumor area while systematically excluding blood vessels, bronchi, and pleura where this was feasible. To minimize heterogeneity bias stemming from variable CT scanners or acquisition parameters, standardization procedures were applied post-segmentation:

(1) gray-level normalization using (window level: -500)/window width 1,500), (2) voxel resampling to 1×1×1 mm³ using B-spline interpolation, and (3) gray-level discretization with a bin width of 25. Subsequent radiomic feature extraction proceeded from the processed ROIs. Radiomic features were extracted using the uAI Research Portal (https://urp.united-imaging.com/; United Imaging Intelligence). The extraction engine of this platform is based on the PyRadiomics framework, which is compliant with the Imaging Biomarker Standardization Initiative (IBSI) standards, ensuring the reproducibility and standardization of the extracted biomarkers

### 2.4 Assessment of radiomic feature reproducibility

To ensure the reliability and stability of the extracted radiomic features, we assessed their inter-observer reproducibility. A subset of 30 patients was randomly selected from the entire cohort. The regions of interest (ROIs) for these patients were independently re-segmented by a second radiologist with over 10 years of experience in thoracic imaging, who was blinded to the initial segmentation results and pathological diagnoses. The Intra-class Correlation Coefficient (ICC) was then calculated using a two-way random-effects model for absolute agreement [ICC (2, 1)] to quantify the consistency of feature values between the two observers. Features with an ICC value greater than 0.75 were considered to have excellent reproducibility and were retained for subsequent analysis, while features with ICC ≤ 0.75 were excluded to minimize variability introduced by segmentation differences.

### 2.5 Radiomic feature selection

**Figure 3:**
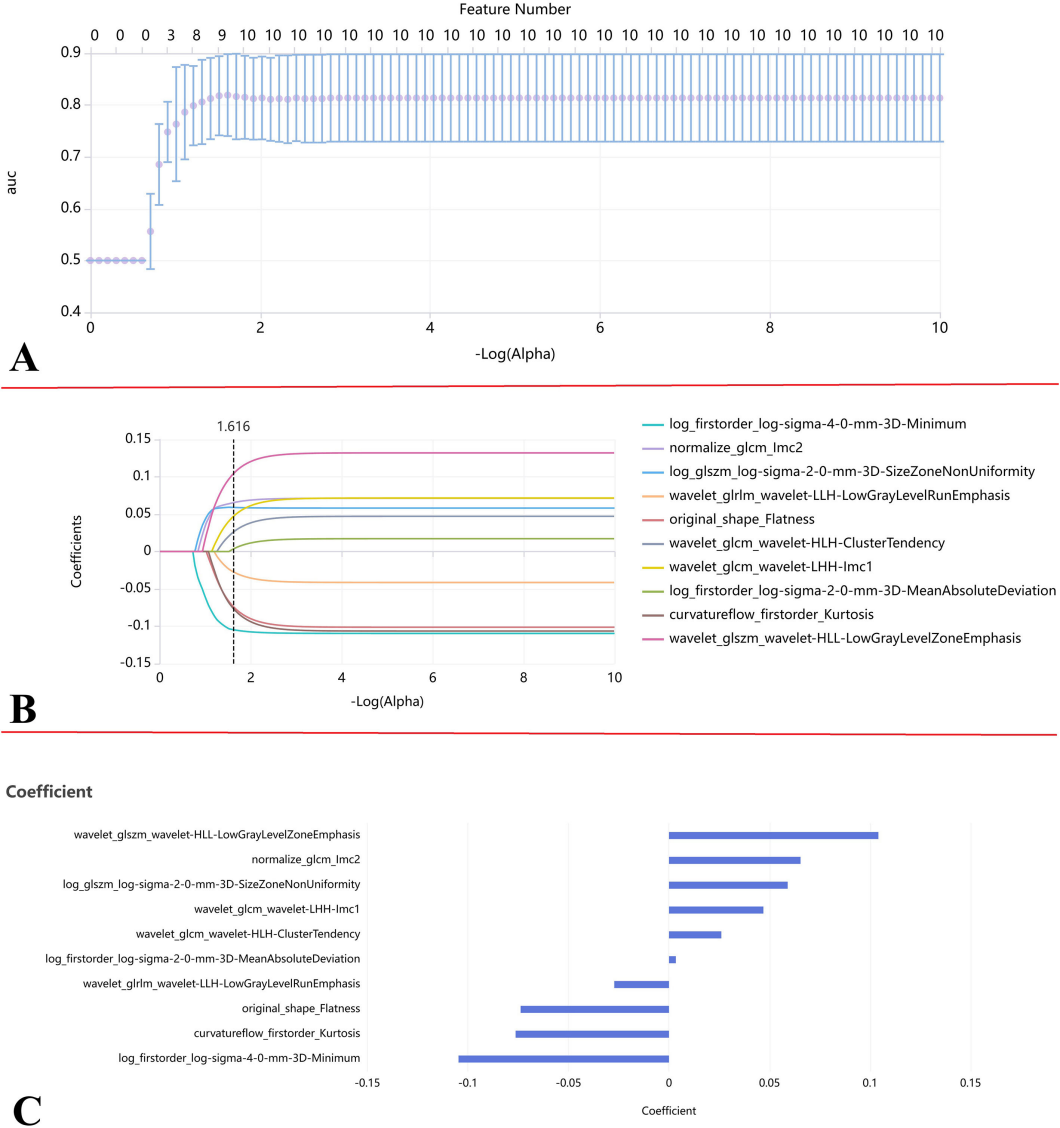
Radiomics feature selection using LASSO logistic regression. **(A)** LASSO coefficient path plot (based on ROI): Ten-fold cross-validation for tunning the LASSO regularization parameter (α). The minimum cross-validation error occurred at log (α)=0.0242, selected as the optimal value. The final LASSO logistic regression model was fitted using this optimal α. **(B)** LASSO coefficient profile plot (based on ROI): Trajectories of the radiomics features coefficients as log (α) decreases. The number of features with non-zero coefficients decrease with increasing regularization strength, demonstrating LASSO’s feature selection property. **(C)** Selected radiomics features after LASSO Screening (based on ROI): The subset of 10 features retained by the LASSO model using the optimal α determined in **(A)**, selected to prevent overfitting.

A total of 2,264 radiomic features were extracted from SSN ROIs, encompassing 14 shape features, 450 first-order features, 400 Grey Level Size Zone Matrix (GLSZM) features, 525 Grey Level Co-occurrence Matrix (GLCM) features, 350 Grey Level Dependence Matrix (GLDM) features, 400 Grey Level Run Length Matrix (GLRLM) features, and 125 Neighborhood Gray-Level Difference Matrix (NGLDM) features. Following reproducibility assessment, 365 features (16.12%) demonstrated good reproducibility (ICC>0.75). Next, the mRMR method was utilized to select the top 14 features with the highest relevance to the pathological invasiveness and the lowest redundancy. Finally, LASSO regressions with 10-fold cross-validation was performed to further refine the feature set. The optimal regularization parameter (λ=0.242) was determined based on the minimum binomial deviance criteria, which ultimately identified 10 key radiomic features for the construction of the signature (**Figure 3**).

### 2.6 Model development​, hyperparameter tuning and validation

Three machine learning classifiers: logistic regression (LR), random forest (RF), and support vector machine (SVM), were trained using the 10-feature signature. To ensure optimal performance and mitigate overfitting, a systematic hyperparameter tuning process was employed via 5-fold cross-validation on the training cohort, which maintained a sufficient number of samples in each validation fold for reliable performance estimation. Separately, a 10-fold cross-validation was chosen for LASSO feature selection to ensure the stability of the penalty parameter (λ) identification.

LR: We utilized the L2 (Ridge) regularization to prevent overfitting. The hyperparameter C, which is the inverse of regularization strength, was optimized. A grid search was performed over a range of values: C = [0.001, 0.01, 0.1, 1, 10, 100].

RF: Key hyperparameters were tuned to balance model complexity and generalization. The optimization process involved: n_estimators: The number of trees in the forest, tested at [50, 100, 200]. max_depth: The maximum depth of the tree, explored with values [3, 5, 7, None]. min_samples_split: The minimum number of samples required to split an internal node, tested at (2, 5, 10). SVM: A linear kernel was selected for the SVM classifier due to its suitability for high-dimensional data, and to maintain model interpretability. The hyperparameter C was optimized over the same range as for LR: [0.001, 0.01, 0.1, 1, 10, 100]. The optimal hyperparameters for each model were defined as the configuration yielding the highest mean AUC during 5-fold cross-validation on the training set. The final models were then refit using the entire training cohort with these optimal parameters. To ensure the methodological rigor and transparency of our radiomics workflow, the study was designed and reported following the Radiomics Quality Score (RQS). The detailed item-by-item calculation is provided in **Supplementary Table 1**. Model performance was evaluated using AUC, sensitivity, specificity, F1 score, ROC curves, calibration curves, precision-recall curves, and DCA.

### 2.7 Model interpretability​ SHAP analysis provided global interpretability by means of a summary plot,

illustrating feature contributions to the LR model predictions. Features were ranked by importance, with colored dots representing individual LUAD patients (yellow = high risk, purple = low risk) across rows corresponding to each feature. This visualization demonstrated how predicted risk levels varied according to feature-specific contributions. Complementary waterfall plots facilitated local interpretability by depicting feature-wise impacts on individual predictions.

### 2.8 Statistical analysis

All analyses were performed using R (v4.1.3). Continuous variables were assessed for normality using Shapiro-Wilk tests and reported as the mean ± standard deviation (normally distributed) or median (interquartile range, IQR) (non-normally distributed). Group comparisons used independent *t*-tests or Mann-Whitney U tests, respectively. Categorical variables were compared using χ² or Fisher’s exact tests. Univariable and multivariable logistic regression identified independent predictors of IAC. Model performance was evaluated using receiver operating characteristic (ROC) curves (area under the curve, AUC), calibration curves (assessing predictive accuracy), precision recall curve (visualizing the tradeoff between precision-positive predictive value and recall sensitivity across different classification thresholds), and decision curve analysis (DCA) to quantify clinical utility. Model discrimination was compared using the DeLong test. Statistical significance was defined as two-sided *p* < 0.05.

## 3 Results

### 3.1 Patient characteristics

The study enrolled 177 patients with LUAD (mean age: 52.24 years ±11.10[SD]; 40 males), who were divided into a training cohort (*n* = 123) and an internal validation cohort (*n* = 54). IAC was pathologically confirmed in 79 patients (44.63%). The baseline characteristics were comparable between cohorts (**Table 1**).

**Table 1:** Clinical characteristics of patients.

Characteristic	Training cohort (n=123)			Validation cohort (n=54)		
	Non-IAC (n=67)	IAC (n=56)	*p* value	Non-IAC (n=31)	IAC (n=23)	*p* value
Gender, n (%)			0.489^a^			0.957 ^a^
Female	57(85.07)	45(80.36)		20(64.52)	15(65.22)	
Male	10(14.93)	11(19.64)		11(35.48)	8(34.78)	
Age, median (IQR)	50.00(18.00)	56.00(13.75)	0.031 ^b^	46.00(19.00)	57.00(20.00)	0.069 ^b^
BMI, median (IQR)	24.14(6.28)	25.61(5.78)	0.178 ^b^	24.34(3.32)	26.35(4.03)	0.019 ^b^
Max diameter, median (IQR)	8.00(2.80)	8.50(3.00)	0.401^b^	8.00(3.00)	9.00(2.00)	0.002 ^b^
Family history of lung cancer			0.372 ^a^			0.426^c^
No	59(88.06)	52(92.86)		28(90.32)	22(95.65)	
Yes	8(11.94)	4(7.14)		3(9.68)	1(4.35)	
Smoking			0.258 ^a^			0.741^a^
No	58(86.57)	52(92.86)		24(77.42)	19(82.61)	
Yes	9(13.43)	4(7.14)		7(22.58)	4(17.39)	
Drinking			0.529 ^a^			1.000^c^
No	62(92.54)	50(89.29)		28(90.32)	20(86.96)	
Yes	5(7.46)	6(10.71)		3(9.68)	3(13.04)	
Location (upper lobe)			0.180^a^			0.975^a^
No	22(32.84)	25(44.64)		19(61.29)	14(60.87)	
Yes	45(67.16)	31(55.36)		12(38.71)	9(39.13)	
Tumor-Lung interface			0.289^c^			0.253^c^
Ill defined	6(8.96)	2(3.57)		3(9.68)	0(0.00)	
Well difined	61(91.04)	54(96.43)		28(90.32)	23(100.00)	
Lobulated sign			0.393 ^a^			
No	29(43.28)	20(35.71)		23(74.19)	10(43.48)	0.022^a^
Yes	38(56.72)	36(64.29)		8(25.81)	13(56.52)	
Spiculated sign			0.294 ^a^			0.052^a^
No	41(59.42)	29(51.79)		24(77.42)	12(52.17)	
Yes	26(40.58)	27(48.21)		7(22.58)	11(47.83)	
VCS			0.015 ^a^			0.055^a^
No	53(79.10)	33(58.93)		25(80.65)	13(56.52)	
Yes	14(20.90)	23(41.07)		6(19.35)	10(43.48)	
Pleural indentation sign			0.847 ^a^			0.724^a^
No	43(64.18)	35(62.50)		19(61.29)	13(56.52)	
Yes	24(35.82)	21(37.50)		12(38.71)	10(43.48)	
Vacuole sign			0.734 ^a^			0.005^a^
No	52(77.61)	42(75.00)		27(87.10)	12(52.17)	
Yes	15(22.39)	14(25.00)		4(12.90)	11(47.83)	

a, Chi-square test; b, Mann-Whiteney U test; c, Fisher Exact Chi-square test.

IAC, Invasive Adenocarcinoma; IQR, ​Interquartile Range; CVS, Convergence Vascular Sign.

### 3.2 Radiomic prediction model development and validation

**Figure 4:**
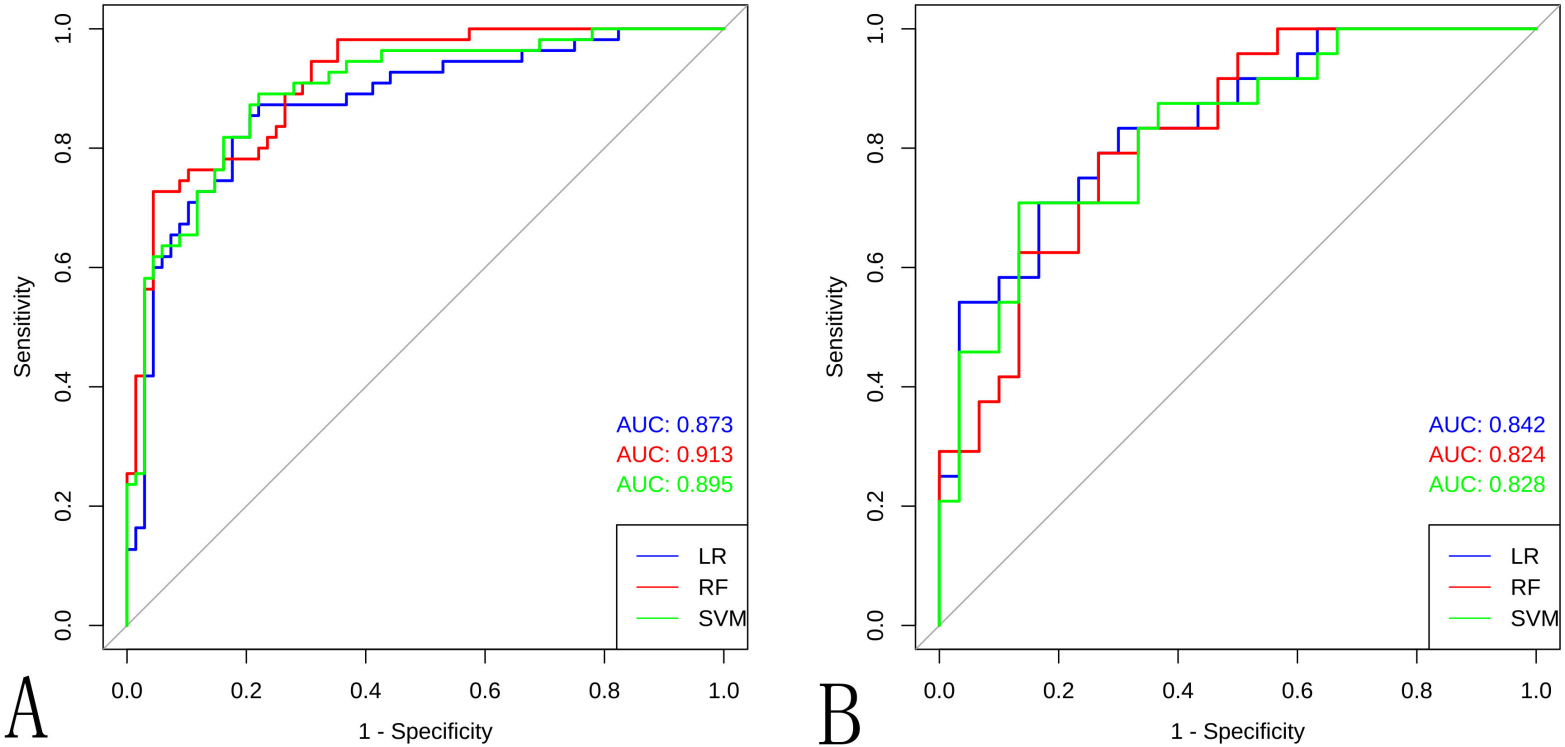
The ROC curve for the different ML models in the **(A)** training cohort and **(B)** internal validation cohort.

**Figure 5:**
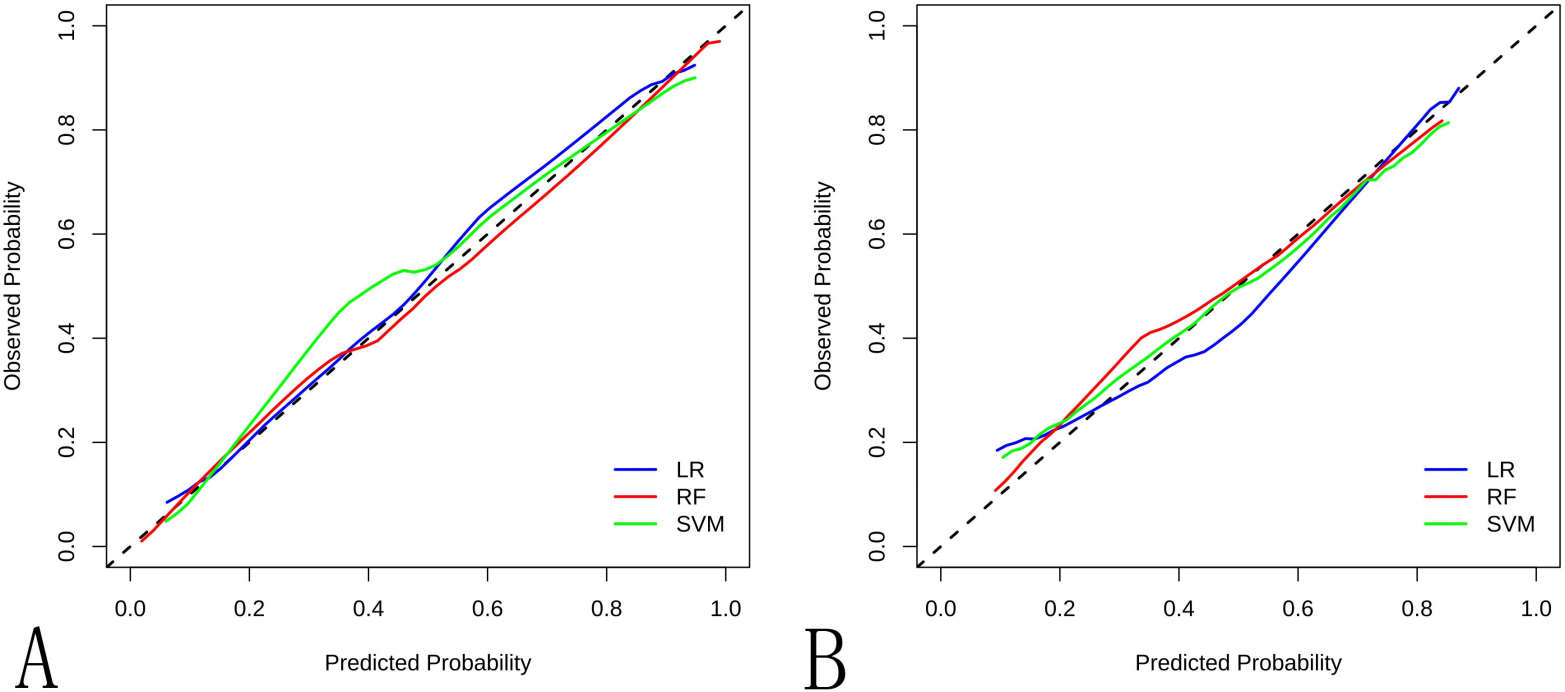
The calibration curve for the different ML models in the **(A)** training cohort and **(B)** internal validation cohort.

**Figure 6:**
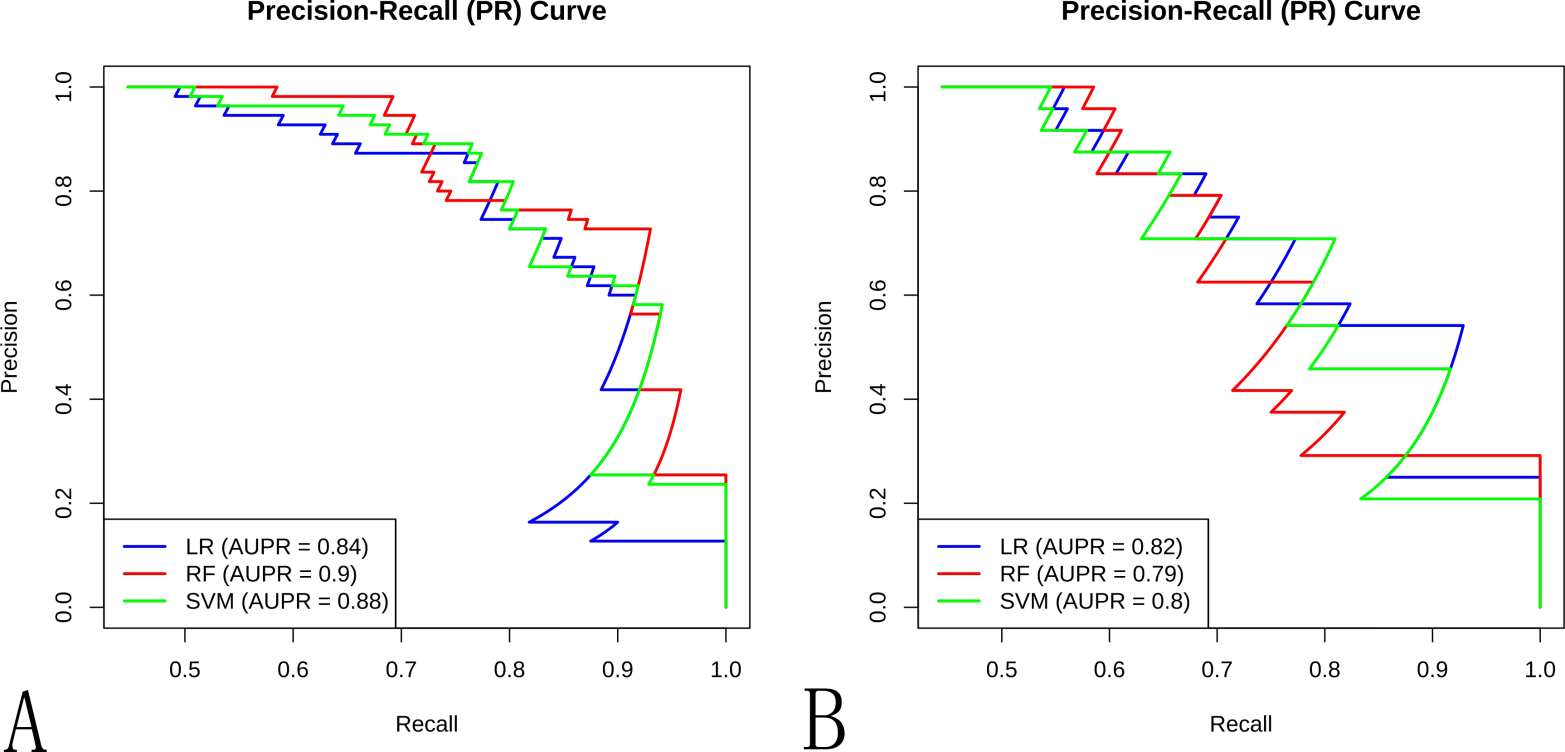
The precision-recall curve of different ML models in the **(A)** training cohort and **(B)** internal validation cohort.

**Figure 7:**
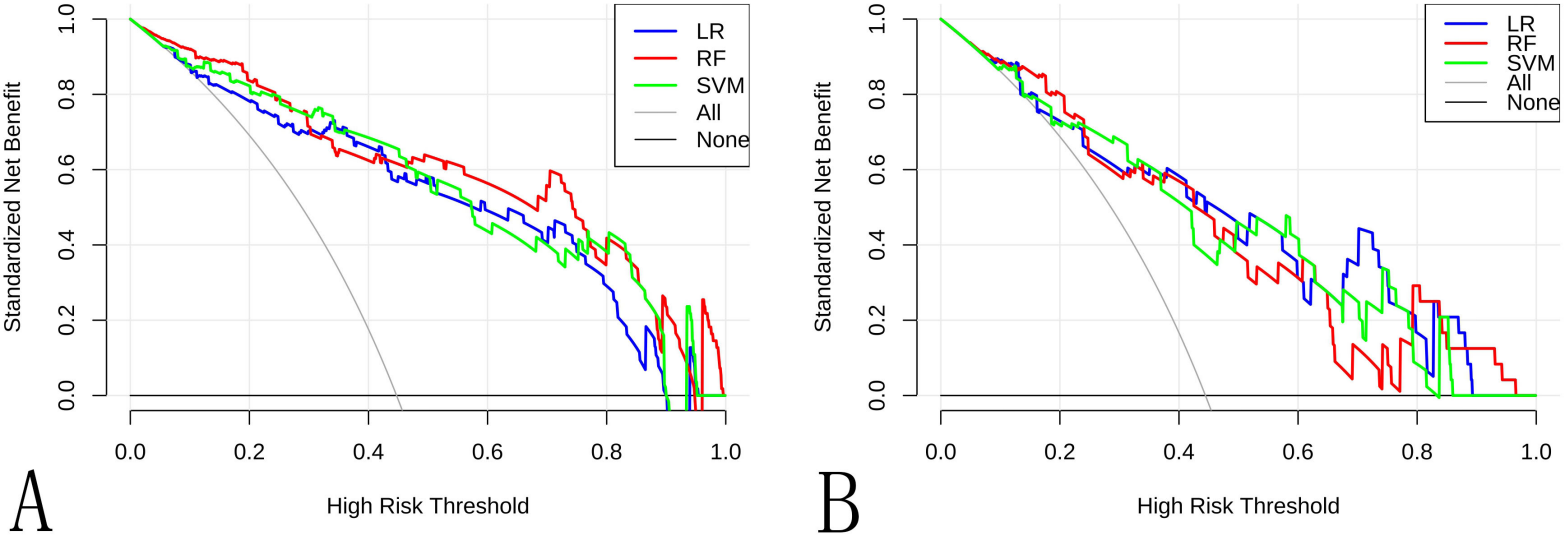
The decision curve analysis for the different ML models in the **(A)** training cohort and **(B)** internal validation cohort showed that the LR provided a higher net benefit than RF, SVM models across most threshold probabilities.

Ten radiomic features were used to train three ML classifiers (LR, RFC, and SVM) to construct and validate the radiomic ML models. Performance metrics of the radiomic models are shown in **Table 2**, **Figures 4**–**7**. To evaluate model stability and mitigate potential overfitting given the cohort size, the performance of all three classifiers was also assessed using 5-fold cross-validation on the entire dataset (n=177). The mean AUCs from the cross-validation were: LR: 0.835 ± 0.055 (95% CI: 0.767-0.903), RF: 0.806 ± 0.066 (95% CI: 0.724-0.888), and SVM: 0.809 ± 0.075 (95% CI: 0.716-0.902). The strong concordance between the cross-validation results and the performance on the held-out internal validation cohort (**Table 2**) underscores the robustness and generalizability of our models, particularly the LR classifier. The LR model was selected as the optimal predictor based on four key criteria: (1) superior generalizability: minimal AUC decline from training to testing (AUC decline: 0.031 vs. RF: 0.089; SVM: 0.068), indicating superior robustness against overfitting. (2) clinically balanced performance: harmonized sensitivity (0.792) and specificity (0.733), mitigating false negative (critical for cancer diagnosis) and false positive risks; (3) robust clinical utility: highest F1 score (0.745) and narrower AUC 95%CI: 0.738–0.946 vs. RF/SVM; (4) compatibility with clinical interpretability frameworks: linear architecture enables transparent SHAP-based explanations. The detailed distribution of correct and incorrect classifications is illustrated in the confusion matrix for the internal test set (**Supplementary Figure 2A**).

**Table 2:** Radiomic model performance.

Methods	AUC (95%CI)		Sensitivity		Specificity		Accuracy		F1 score	
	Train	Test	Train	Test	Train	Test	Train	Test	Train	Test
LR	0.873(0.809-0.938)	0.842(0.738-0.946)	0.727	0.792	0.868	0.733	0.805	0.759	0.769	0.745
RF	0.913(0.864-0.961)	0.824(0.715-0.932)	0.891	0.833	0.721	0.600	0.797	0.704	0.797	0.714
SVM	0.896(0.839-0.952)	0.828(0.718-0.937)	0.727	0.708	0.708	0.853	0.797	0.685	0.762	0.667

LR, Logistic Regression; RF, Random Forest; SVM, Support Vector Machine; AUC, Area Under Curve.

### 3.3 Statistical comparison of model performance

To objectively evaluate the relative performance of the classifiers, DeLong’s test was used to compare their ROC curves derived from the internal validation cohort. The results of these pairwise comparisons are summarized in **Table 3**. No statistically significant difference in AUC was found between the top-performing LR model and the RF model (p = 0.589). Similarly, the difference in AUC between the LR and SVM models was not statistically significant (p = 0.372). The comparison between RF and SVM also yielded a non-significant result (p = 0.889).

**Table 3:** Statistical comparison of classifier performance in the validation cohort using DeLong’s test.

Comparison (validation cohort)	Z	p value
LR vs. RF	0.540	0.5889
LR vs. SVM	0.893	0.3716
RF vs. SVM	0.140	0.8888

### 3.4 Performance in the external validation cohort

**Figure 8:**
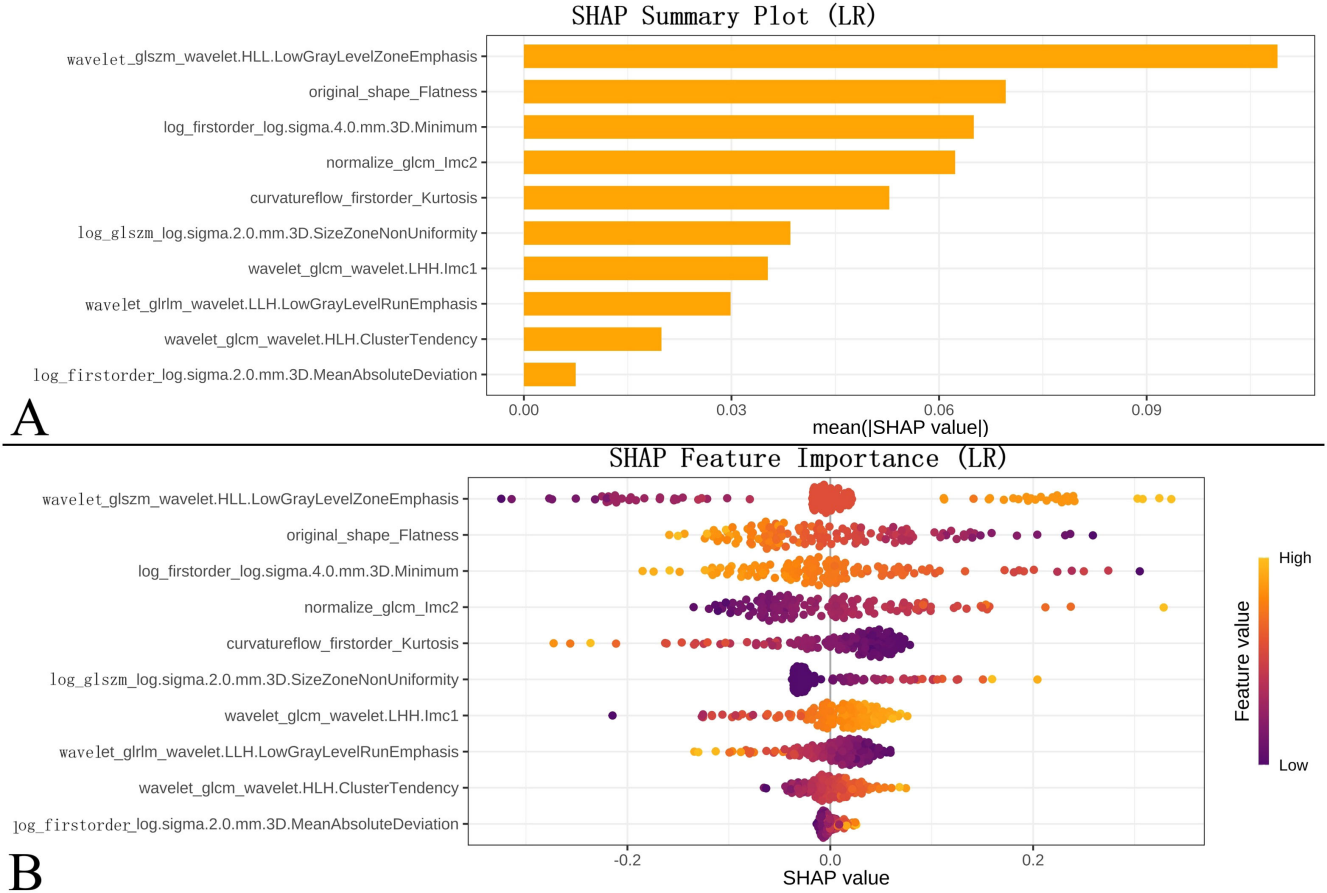
Model interpretability using SHAP. **(A)** SHAP bar chart: Feature importance ranking based on mean absolute SHAP values. **(B)** SHAP bees warm plot: Distribution of feature impacts on model output (prediction probability); yellow/purple indicate higher/lower feature values, respectively.

The optimal model (LR) was tested on the external cohort to evaluate its institutional robustness. Despite being trained on Center 1 data, the model achieved an AUC of 0.778, with a sensitivity of 73.91% and specificity of 71.67% (**Figure 8**). To further visualize the model’s diagnostic accuracy across centers, the confusion matrix for the external validation cohort is presented in **Supplementary Figure 2B**. These results, presented via independent ROC, PRC, and DCA curves (**Supplementary Figure 1**), demonstrate that our radiomics-ML pipeline is highly effective when applied to external data acquired under standardized scanning conditions.

### 3.5 Model interpretation

**Figure 9:**
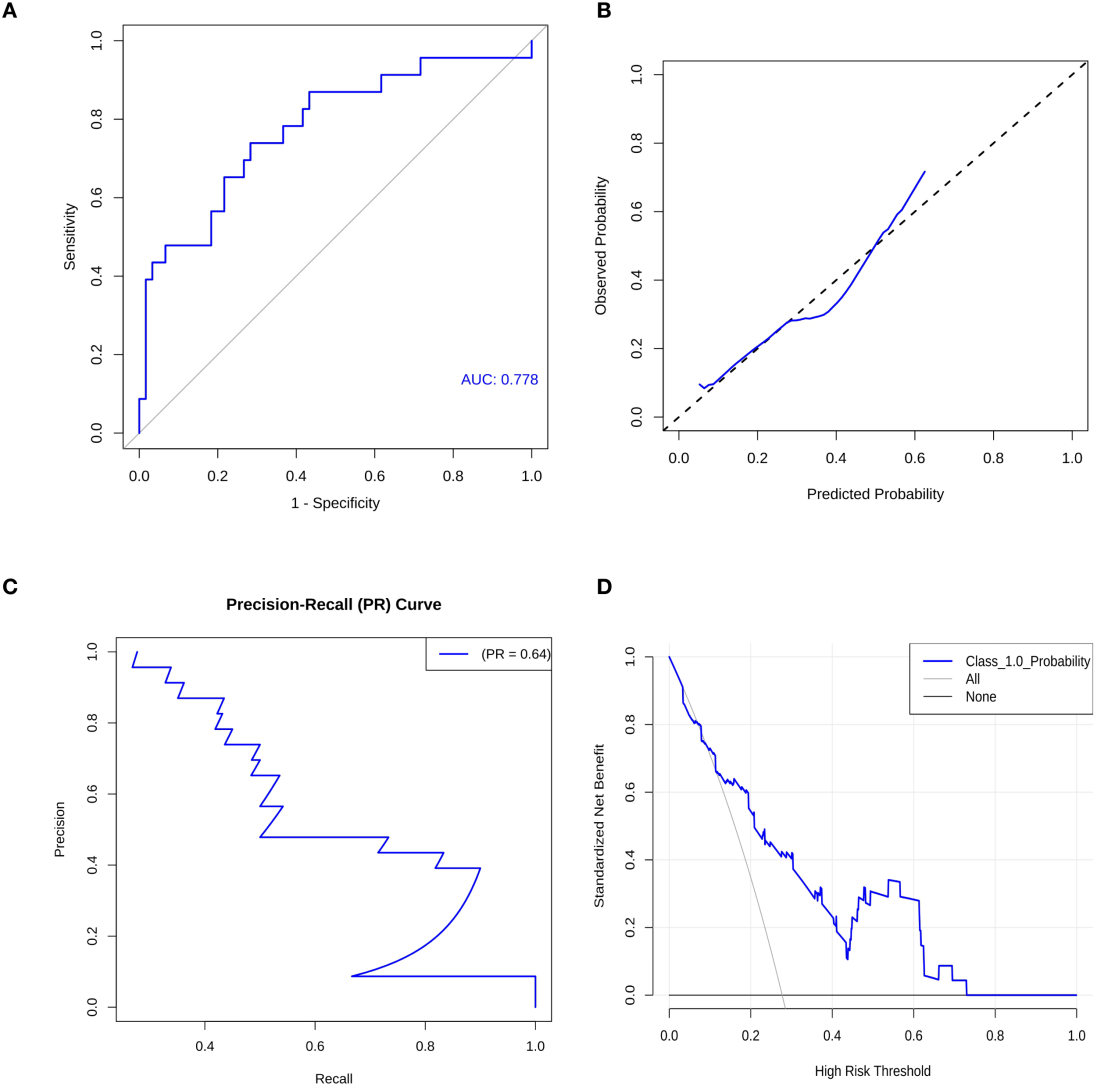
Performance evaluation of the logistic regression (LR) model in the external validation cohort. **(A)** Receiver operating characteristic (ROC) curve demonstrating the discriminative ability of the model (AUC = 0.778). **(B)** Calibration curve illustrating the agreement between predicted and observed probabilities of invasiveness. **(C)** Precision-recall (PR) curve showing the trade-off between precision and recall (PR = 0.64). **(D)** Decision curve analysis (DCA) evaluating the clinical net benefit of the model across a range of threshold probabilities.

**Figure 10:**
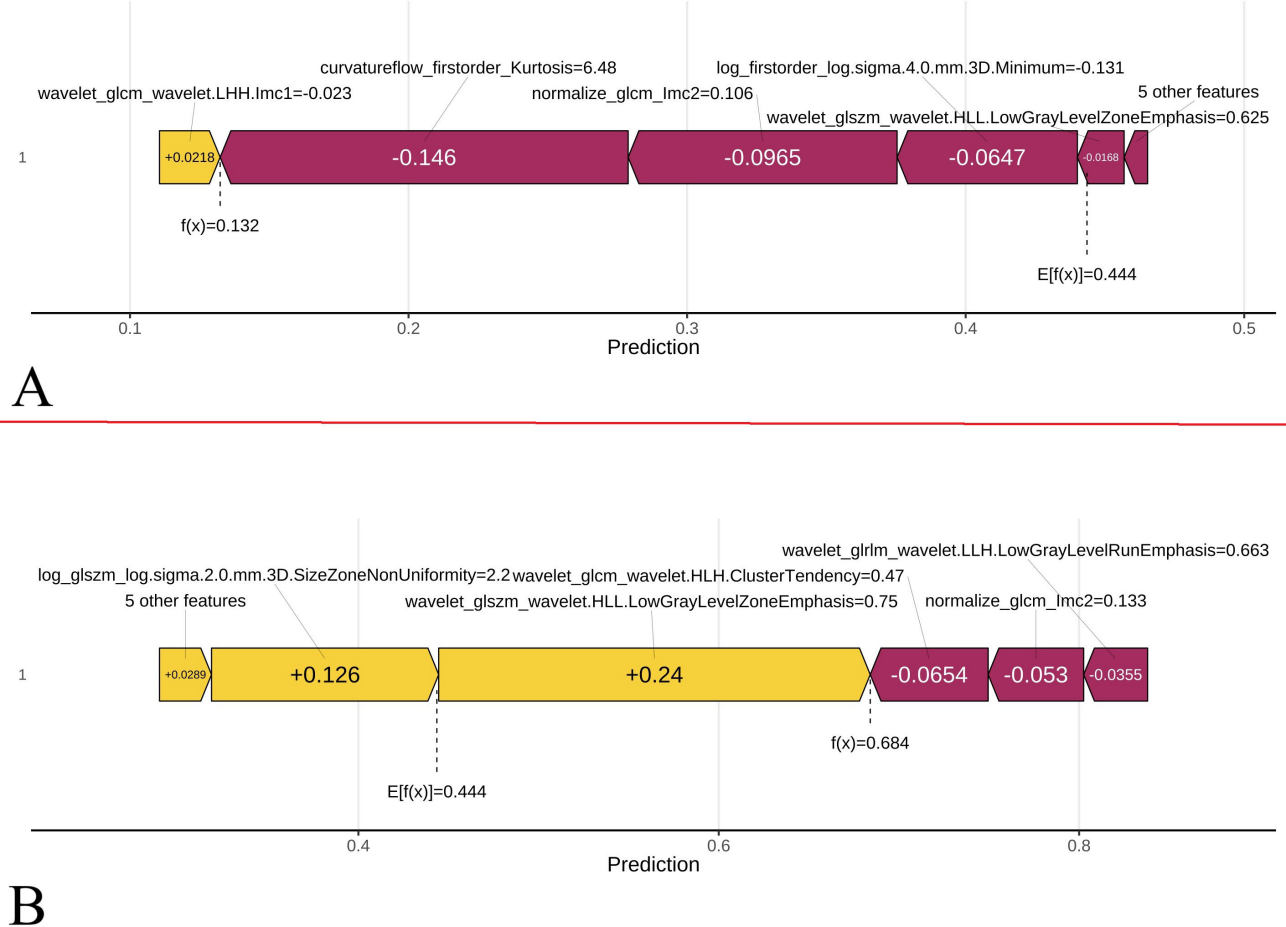
SHAP force plots illustrating the contribution of individual features to the final prediction probability for two representative cases. SHAP values elucidated personalized risk profiles, aiding clinicians in tailored treatment planning. **(A)** A 34-year-old female patient with minimally invasive adenocarcinoma (MIA), showing a lower predicted probability of invasiveness. **(B)** A 50-year-old male patient with invasive adenocarcinoma (IAC), showing a higher predicted probability of invasiveness.

SHAP analysis was performed to quantify feature importance and provide both global and local model interpretability (**Figure 9**). The top predictive features were ranked by their mean absolute SHAP values in a bar chart (**Figure 9A**), while a beeswarm plot was utilized to illustrate the directionality of feature impacts, where yellow and purple dots represent higher and lower feature values, respectively (**Figure 9B**). Additionally, force plots were generated to visualize feature-level contributions to individual predictions (**Figure 10**). Through this analysis, SHAP identified wavelet_glszm_wavelet.HLL.LowGrayLevelZoneEmphasis, original_shape_Flatness, and log_firstorder_log.sigma.4.0.mm.3D.Minimum as the top discriminative features.

## 4 Discussion

Our study addresses two critical gaps in the radiomics literature on pulmonary nodules. First, we specifically targeted the diagnostic challenge of SSNs—a population increasingly detected by LDCT screening yet notably underrepresented in prior research, which often prioritized larger, solid lesions (16, 17). The management of these minute nodules remains highly controversial due to the limitations of subjective CT assessment (18, 19), creating a pressing unmet clinical need. Second, and more importantly, we moved beyond the common “black-box” paradigm by making model interpretability a cornerstone of our approach. Through the comprehensive integration of SHAP analysis, we not only predict invasiveness but also elucidate the underlying reasoning by identifying the most predictive features and explaining their directionality and potential biological significance. This commitment to transparency is a critical step toward building clinical trust and facilitating the integration of artificial intelligence tools into real-world decision-making processes (20, 21).

Our findings directly address the limitations of conventional CT assessment for SSNs (16, 22, 23). Although qualitative features such as vacuoles and vascular convergence showed statistical association with invasiveness in univariate analysis (**Table 1**), their diagnostic consistency was inadequate across cohorts. Radiomics objectively quantified an otherwise imperceptible tumor heterogeneity, yielding significantly improved diagnostic precision. The robust performance of the LR model (AUC 0.842) underscores radiomics’ potential to augment subjective evaluation, particularly for nodules displaying equivocal morphological features. The LR model in our study demonstrated balanced and robust performance (AUC: 0.842) in the internal validation, a result comparable to the advanced models reported for larger SSNs, such as the deep-learning-assisted nomogram by Zuo et al. (12) and the stacking ensemble classifier by Li et al. (13). This indicates that a well-constructed radiomics model can achieve high diagnostic accuracy without necessarily resorting to complex architectures. A critical finding from our statistical comparison was that DeLong’s test confirmed no significant difference in AUC between the top-performing LR model and the RF or SVM models. This statistical equivalence is pivotal, as it shifts the model selection criteria from pure discriminatory power to a holistic evaluation of generalizability, stability, and clinical applicability. Consequently, the selection of the LR model is strongly justified by its optimal balance of three factors. First, it exhibited superior generalizability, evidenced by the minimal AUC decline from training to validation (ΔAUC 0.031), which was relatively less than that of RF (0.089) or SVM (0.068), demonstrating robust resistance to overfitting (24, 25). Second, it achieved a clinically essential equilibrium between sensitivity (0.792) and specificity (0.733), effectively mitigating the risks of both under-treatment and unnecessary intervention, as further validated by its high F1 score (0.745) (26). Finally, and crucially, unlike complex “black-box” models, LR’s linear architecture provides an inherently transparent foundation (27), which we seamlessly integrated with SHAP analysis. This integration moves beyond mere prediction to explain the “why” behind each decision, providing both global and local explanations that are fundamental for building clinician trust and fostering clinical adoption (28). This combination of non-inferior predictive power, proven stability, and inherent explainability makes LR the most clinically pragmatic choice for deployment. A notable strength of our study is the implementation of hardware-level standardization for the external validation. By selecting an external cohort scanned with identical CT models and default parameters, we successfully isolated the biological and pathological variations from potential technical artifacts. The stable performance of the LR model across both centers suggests that our radiomics signatures are intrinsically linked to the tumor’s invasive potential rather than specific imaging noise.

The integration of SHAP analysis, a core novelty of our work, yielded unprecedented insights into the biological mechanisms captured by our model. The top-ranked features suggested potential links to tumor pathobiology: wavelet_HLL_glszm_LowGrayLevelZoneEmphasis: This feature quantified the spatial clustering of low-attenuation voxels in the wavelet-transformed HLL (horizontal-high, vertical-low, diagonal-low) sub-band. Higher values indicated larger contiguous regions of low CT attenuation (-600 to -800 HU). In IAC, aggressive tumor behaviors induced necrotic zones from rapid growth exceeding angiogenesis and microcystic fusion due to parenchymal collapse. These processes created macroscale low-attenuation clusters that were visible in the HLL sub-band, which emphasized horizontal structural disruptions. Consequently, elevated feature values correlated with advanced invasiveness (29).

Original_shape_Flatness: this radiomic feature measured the three-dimensional (3D) planarity or flatness of an ROI, with values on a scale of 0 to 1. Higher values (approaching 1) indicated a more planar or isotropic shape, geometrically approximating a cube or sphere. Conversely, lower values (approaching 0) corresponded to a more elongated, rod-like morphology. This feature primarily described the 3D geometric properties of the ROI, specifically its spatial directional dimensionality and deviation from sphericity. This may reflect the ROI’s growth pattern and interactions with surrounding tissues.

Evidence indicates that morphological features, including flatness, serve as effective differentiators between lung adenocarcinoma pathological subtypes (AIS/MIA vs. IAC) (30–32). The characteristically more irregular morphology of IAC, stemming from its aggressive growth patterns (destructive growth, complex structures such as micropapillary/solid components, and infiltration along structures), could manifest through alterations in flatness: (1) a decrease in overall flatness (suggesting a trend towards elongation), and (2) localized increases in flatness in specific areas (e.g., flat patches along the pleura). Critically, an overall increase in morphological complexity represented a more critical marker of invasiveness.

The log_firstorder_log.sigma.4.0.mm.3D_Minimum: this feature represented the minimum intensity value after Laplacian of Gaussian (LoG) filtering at σ=4mm scale. Lower values (more negative) indicated extremely low-attenuation foci (~-900 to -1000 HU). In pre/minimally invasive lesions (AIS/MIA), preserved alveolar airspaces generate isolated voxels with near-air density, and “bubble-like” pseudo-cavitations formed due to lepidic growth without destruction. The LoG filter (σ=4mm) amplified these submillimeter air-trapping zones. As invasiveness increased, tumor cells filled airspaces, reducing extreme low-attenuation voxels. Thus, lower minimum values (more negative) predicted lower invasiveness. Consistent with the findings of Zuo et al. (12). and Li et al. (13)., our SHAP analysis also identified features related to tumor morphology and patient demographics as top contributors. Specifically, the importance of features like wavelet_HLL_glszm_LowGrayLevelZoneEmphasis (potentially indicating necrosis) and original_shape_Flatness (reflecting morphological irregularity) in our model aligns with the principle that radiomics captures critical tumor heterogeneity. The concordance in key predictors, such as morphological complexity, across independent studies strengthens the biological plausibility of radiomic models. These SHAP-derived insights transcended simple feature lists, elucidating how specific quantitative image characteristics mechanistically contributed to invasiveness prediction. Future histopathological correlation studies linking these features to specific tumor microenvironment characteristics (e.g., fibroblast proliferation, architectural distortion, microvascular patterns) are essential.

The “black box” nature of numerous ML models remains a significant barrier to clinical adoption (15). Our integration of SHAP explicitly addressed this limitation. Global summaries provided clinicians with key radio-phenotypic determinants of SSN invasiveness, potentially refining human pattern recognition. Local explanations using force plots personalize predictions for individual patients and visually delineate the contribution of each feature towards the nodule-specific risk score. This transparency facilitated clinician-patient communication, supported tailored clinical decision-making (e.g., surgical planning versus active surveillance), and cultivated trust among radiologists and clinicians (33, 34). Furthermore, DCA validated the model’s net clinical benefit over a broad range of threshold probabilities, highlighting its practical advantage over binary treat-all or treat-nothing strategies. In terms of methodological rigor, our study design adheres to the core recommendations of the Radiomics Quality Score (RQS) framework (detailed in **Supplementary Table 1**). By implementing rigorous feature stability screening (ICC > 0.75), multi-step dimensionality reduction, and, importantly, independent external validation, we have endeavored to ensure the reproducibility and clinical relevance of our radiomic signature.

This study has several limitations that warrant consideration. First, although we successfully incorporated an independent external validation cohort, the retrospective nature of the study may still introduce selection bias. Since the model was developed and validated using data from a single institution with specific CT scanners and imaging protocols, the performance and stability of both the radiomic features and the predictive model may vary when applied to external cohorts with different acquisition parameters and demographic characteristics. Second, although SHAP analysis improves interpretability, the biological plausibility of the top radiomic features—while informed by existing literature—remains hypothetical and requires further validation through histopathological correlation. Future studies incorporating precise region-of-interest matching between CT imaging and histopathological samples (e.g., via tissue microarrays or detailed pathologic mapping) are necessary to confirm the underlying biological mechanisms of these image-based biomarkers. Third, to ensure model parsimony and mitigate overfitting risks given the sample size, clinical variables (e.g., age, smoking history) and semantic radiological features were not incorporated. Their inclusion in future iterations may improve model comprehensiveness and predictive power. Third, due to the retrospective nature of the data collection, we were unable to explicitly evaluate the performance differences between the specific CT scanner models (GE and Philips) used in our primary cohort. Although we applied rigorous IBSI-recommended image preprocessing (e.g., 1×1×1 mm³ spatial resampling and gray-level normalization) to minimize inter-scanner variability prior to feature extraction, future prospective studies should track specific hardware parameters to conduct comprehensive inter-vendor subgroup analyses. Finally, although logistic regression was selected for its favorable performance and interpretability, more complex yet explainable approaches—such as explainable boosting machines or deep learning-based radiomic models—may capture subtler patterns and should be investigated in larger, multi-center datasets.

While our study has been strengthened by the inclusion of an independent external validation cohort from a second center, it remains a retrospective analysis. Future prospective, large-scale multicenter studies are still warranted to further confirm the model’s robustness and clinical utility across more diverse clinical settings and imaging platforms prior to widespread implementation.

## 5 Conclusions

This study establishes a robust and interpretable radiomics-LR model for invasiveness prediction in subcentimeter SSNs. By combining rigorous feature extraction, ML validation, and SHAP explanations, this tool provides biological insights and clinical decision support. Prospective multi-center validation is warranted for clinical translation.

## Data Availability

The original contributions presented in the study are included in the article/**Supplementary Material**. Further inquiries can be directed to the corresponding author.
