# Supplementary material for: Radiomics-machine learning model for predicting invasiveness of subcentimeter subsolid lung adenocarcinoma: a validation study with external cohort and SHAP interpretability
Source: Front Oncol. 2026 Mar 26;16:1668102. doi: 10.3389/fonc.2026.1668102 (PMC13062904; doi:10.3389/fonc.2026.1668102)
Supplement: Supplementary file 1 [file DataSheet1.docx]

**Table S1.** Detailed Assessment of the Radiomics Quality Score (RQS) and Radiomics Reporting Guidelines (RRL) for the Present Study.

| RRL | Criteria | Points |  | RRL |  | Points |
| --- | --- | --- | --- | --- | --- | --- |
| RRL1 | 1.Unmet clinical need | 2 |  | RRL5 | 23. Comparison with previous work | 1 |
| RRL1 | 2. Hardware description | 1 |  | RRL5 | 24. Potential clinical utility | 2 |
| RRL1 | 3. Image protocol quality | 2 |  | RRL6 | 25.Explainability | 1 |
| RRL1 | 4.Inclusion and exclusion criteria | 1 |  | RRL6 | 26.Evaluation of explainability | 1 |
| RRL1 | 5. Diversity and distribution | 1 |  | RRL6 | 27.Biological correlates | 1 |
| RRL2 | 6.Fuature robustness | 1 |  | RRL6 | 28.Evaluation of fairness and plan for mitigation of bias | 1 |
| RRL2 | 7.Preprocessing of image | 1 |  | RRL7 | 29.Usablitiy for clinicians | 0 |
| RRL2 | 8.Harmonization | 1 |  | RRL7 | 30.Sample size calcution | 0 |
| RRL2 | 9.Compliance with international standards in radiomics | 1 |  | RRL7 | 31.Clinical trial preregistration | 0 |
| RRL2 | 10.Automatic segmentation | 1 |  | RRL7 | 32.Prospective validation | 0 |
| RRL3 | 11.Feature reduction | 1 |  | RRL7 | 33.Real-world clinical assessment | 0 |
| RRL3 | 12.Feature robustness for feature selection | 1 |  | RRL8 | 34.Software traceability | 1 |
| RRL3 | 13.Combination of HCR and DLR | 0 |  | RRL8 | 35.Software safeguard | 1 |
| RRL3 | 14.Multivariable analysis | 2 |  | RRL8 | 36.Cost-effectiveness analysis | 0 |
| RRL4 | 15.Single-centre validation | 1 |  | RRL8 | 37.Performance drift | 0 |
| RRL4 | 16.Cut-off analysis | 1 |  | RRL8 | 38.Continual learning | 0 |
| RRL4 | 17.Discrimination statistics | 1 |  | RRL9 | 40.Quality management system | 1 |
| RRL4 | 18.Calibraion statistics | 1 |  | RRL9 | 41.Regulatory requirement | 0 |
| RRL4 | 19.Faiure mode analysis | 0 |  | RRL9 | 42.Product on the market | 0 |
| RRL4 | 20.Open science and data | 0 |  | RRL9 | 43. |  |
| RRL5 | 21.Multicentre validation | 1 |  |  |  |  |
| RRL5 | 22.Comparsion with current clinical standard | 2 |  |  |  |  |
