# Supplementary figures and images for: Radiomics-machine learning model for predicting invasiveness of subcentimeter subsolid lung adenocarcinoma: a validation study with external cohort and SHAP interpretability
Source: Front Oncol. 2026 Mar 26;16:1668102. doi: 10.3389/fonc.2026.1668102 (PMC13062904; doi:10.3389/fonc.2026.1668102)

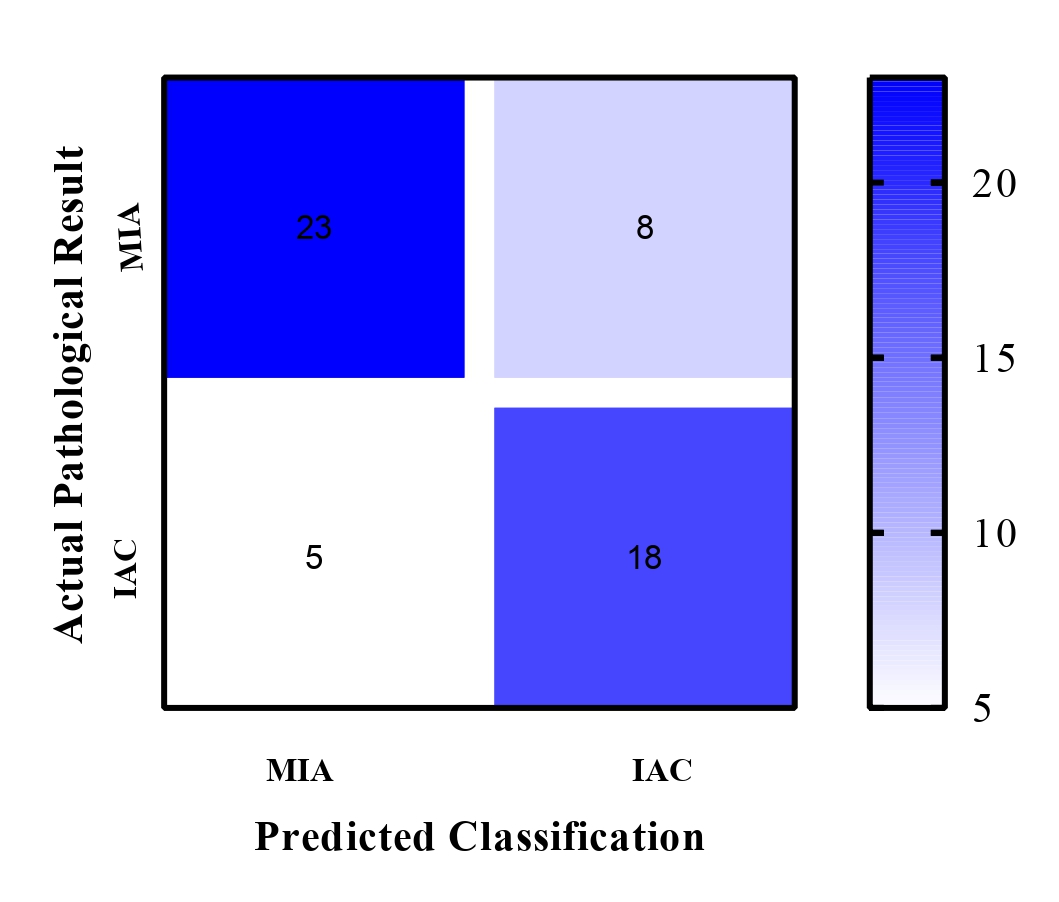

Supplement: Supplementary file 2 [file Image1.jpeg]

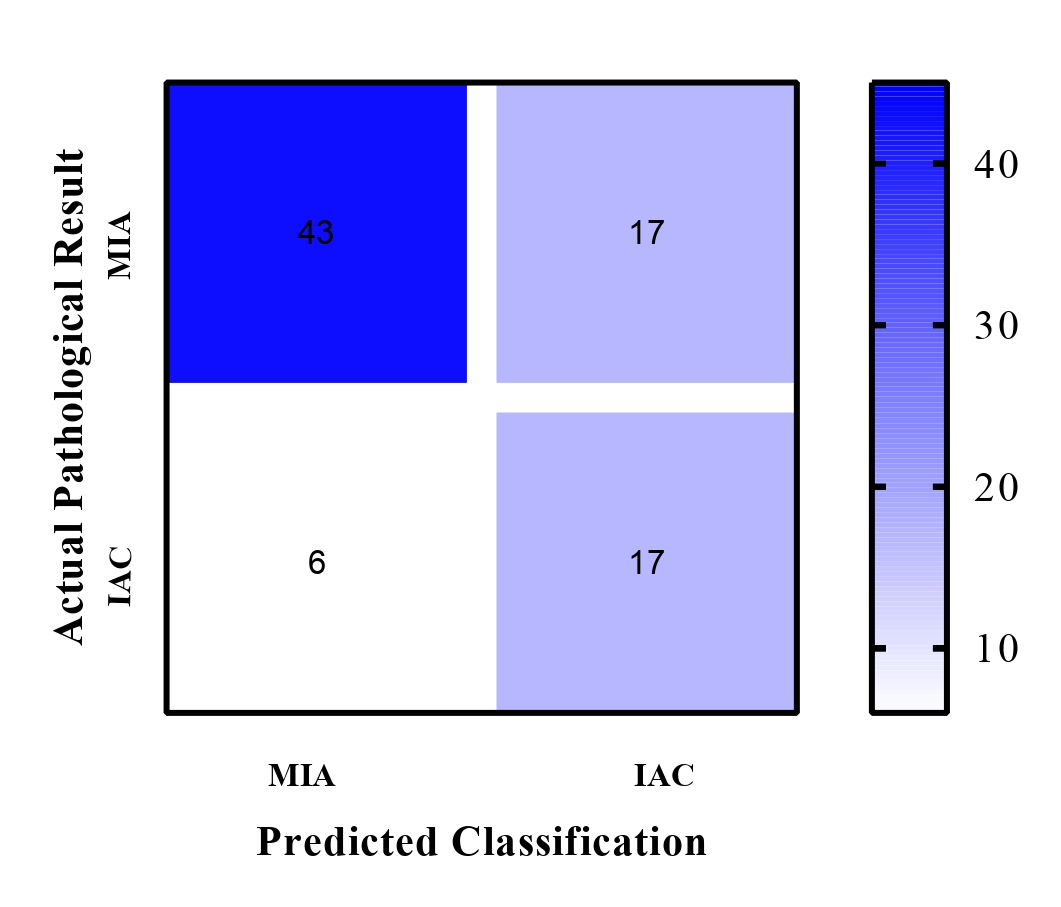

Supplement: Supplementary file 3 [file Image2.jpeg]
